# The diagnostic significance of neutrophil-to-lymphocyte ratio and platelet-to-lymphocyte ratio in malaria: insights and implications – a narrative review

**DOI:** 10.1097/MS9.0000000000003156

**Published:** 2025-03-20

**Authors:** Emmanuel Ifeanyi Obeagu

**Affiliations:** Department of Biomedical and Laboratory Science, Africa University, Mutare, Zimbabwe

**Keywords:** diagnostic biomarkers, immune response, malaria, neutrophil-to-lymphocyte ratio, platelet-to-lymphocyte ratio

## Abstract

Malaria remains a significant global health challenge, necessitating accurate diagnostic tools for timely intervention and effective management. This review explores the diagnostic significance of two hematological biomarkers, the neutrophil-to-lymphocyte ratio (NLR) and platelet-to-lymphocyte ratio (PLR), in the context of malaria. These ratios, derived from routine complete blood count parameters, offer insights into systemic inflammation and immune response dynamics, contributing valuable information for assessing disease severity and predicting clinical outcomes. The NLR and PLR have emerged as promising indicators of malaria severity, reflecting the host’s immune response and inflammatory state during infection. Elevated NLR levels are associated with severe malaria manifestations, including cerebral involvement and organ dysfunction, indicative of dysregulated immune activation characterized by heightened neutrophil activity and lymphocyte depletion. Conversely, alterations in PLR suggest platelet consumption and immune-mediated thrombocytopenia, which correlate with disease progression and complications. Integrating NLR and PLR into malaria diagnostic algorithms holds potential benefits for clinical practice, particularly in resource-limited settings where access to sophisticated diagnostic tools may be constrained. Rapid calculation from standard complete blood count results facilitates timely risk assessment and treatment decision-making, complementing existing diagnostic methods like rapid diagnostic tests and microscopy. Furthermore, longitudinal monitoring of NLR and PLR dynamics during treatment provides valuable insights into therapeutic response and disease resolution, aiding in patient management strategies aimed at optimizing outcomes and reducing mortality rates associated with severe malaria.

## Introduction

Malaria continues to pose a significant global health burden, particularly affecting populations in tropical and subtropical regions^[1]^. Caused by Plasmodium parasites transmitted through the bites of infected Anopheles mosquitoes, malaria manifests with varying severity, ranging from mild flu-like symptoms to life-threatening complications such as cerebral malaria and multi-organ failure. Despite advances in preventive measures and treatment options, malaria remains a leading cause of morbidity and mortality worldwide, with an estimated 229 million cases and 409 000 deaths reported in 2019 alone^[2]^. Accurate and timely diagnosis is crucial for effective malaria management, enabling prompt initiation of appropriate treatment and prevention of severe outcomes^[3]^. While rapid diagnostic tests (RDTs) and microscopic examination of blood smears are primary diagnostic modalities, they may have limitations in sensitivity, especially in detecting low-level parasitemia or differentiating between species of Plasmodium^[4]^. Therefore, there is a continuous need for adjunctive biomarkers that can enhance diagnostic accuracy, provide insights into disease severity, and predict treatment outcomes. Hematological biomarkers such as the NLR and PLR have garnered increasing attention for their potential diagnostic and prognostic value in infectious diseases and inflammatory conditions^[5]^. These ratios, derived from routine complete blood count (CBC) parameters, reflect systemic inflammation and immune response dynamics. In malaria, NLR and PLR offer additional insights into host immune activation and pathological processes beyond parasitological measures, presenting an opportunity to refine diagnostic algorithms and improve clinical decision-making.

**HIGHLIGHTS**

- NLR and PLR offer cost-effective, accessible biomarkers for malaria diagnosis.
- Elevated NLR correlates with severe malaria and poor outcomes.
- PLR reflects platelet consumption and immune-mediated thrombocytopenia.
- NLR and PLR enhance traditional diagnostic methods.
- Future research needed for standardized cutoffs and clinical integration.

The NLR, defined as the ratio of absolute neutrophil count to absolute lymphocyte count, serves as a marker of systemic inflammation and stress response^[6]^. Elevated NLR has been associated with various infectious diseases and inflammatory states, correlating with disease severity and poor clinical outcomes. In the context of malaria, elevated NLR levels have shown promise in predicting severe manifestations such as cerebral malaria and acute respiratory distress syndrome (ARDS), highlighting its potential utility as a prognostic indicator and therapeutic monitoring tool. Similarly, the PLR, calculated as the ratio of platelet count to lymphocyte count, reflects alterations in platelet homeostasis and immune-mediated platelet consumption^[7]^. Thrombocytopenia is a common hematological manifestation in malaria, attributed to both direct parasite-induced destruction of platelets and immune-mediated mechanisms. Changes in PLR during malaria infection may thus provide insights into disease progression, complications like disseminated intravascular coagulation, and response to anti-malarial treatment. The integration of NLR and PLR into clinical practice holds promise for enhancing the diagnostic accuracy and prognostic stratification of malaria cases, particularly in resource-limited settings where access to advanced diagnostic tools may be limited^[8]^. Rapid calculation of these ratios from routine CBC results offers a cost-effective and accessible method to complement traditional diagnostic approaches like RDTs and microscopy. Furthermore, longitudinal monitoring of NLR and PLR dynamics during the course of malaria treatment could provide valuable insights into treatment response, disease resolution, and the emergence of complications, guiding personalized therapeutic strategies tailored to individual patient needs.

Despite their potential benefits, challenges remain in standardizing cutoff values, interpreting ratios in the context of coexisting infections or comorbidities, and integrating NLR and PLR into existing malaria management guidelines^[9]^. Additionally, variations in demographic factors, malaria endemicity, and parasite species may influence the utility of these biomarkers across different populations. Addressing these challenges requires robust clinical validation studies, collaborative research efforts, and consensus guidelines to optimize the clinical utility and global applicability of NLR and PLR in malaria diagnostics and patient management. This review aims to comprehensively examine the diagnostic significance of NLR and PLR in malaria, exploring their roles as adjunctive biomarkers for assessing disease severity, predicting clinical outcomes, and monitoring treatment response.

## Aim

The aim of this review is to comprehensively evaluate the diagnostic significance of the NLR and PLR in malaria. Specifically, the review seeks to:

- Investigate the potential of NLR and PLR as biomarkers for assessing disease severity and immune response in malaria.
- Explore the correlation between NLR and PLR values with clinical outcomes in malaria patients, including complications and mortality.
- Assess the utility of NLR and PLR in complementing traditional diagnostic methods such as RDTs and microscopy.
- Identify the challenges and limitations associated with the use of NLR and PLR in clinical practice, including variability in cutoff values and the influence of comorbid conditions.
- Provide recommendations for future research and clinical applications of NLR and PLR in the context of malaria diagnosis and management, particularly in resource-limited settings.

## Rationale

Malaria remains a significant global health challenge, particularly in tropical and subtropical regions where it causes substantial morbidity and mortality. Effective management of malaria relies on accurate and timely diagnosis to ensure appropriate treatment and prevent severe complications. Traditional diagnostic methods, such as RDTs and microscopic examination of blood smears, while essential, have limitations in sensitivity and specificity, particularly in detecting low-level parasitemia and distinguishing between different species of Plasmodium. As a result, there is a continuous need for adjunctive biomarkers that can enhance diagnostic accuracy, assess disease severity, and predict treatment outcomes. The NLR and PLR are emerging as promising hematological biomarkers that reflect systemic inflammation and immune response dynamics. Derived from routine CBC parameters, these ratios offer a cost-effective and accessible means of providing additional insights into the host’s immune status during malaria infection. Elevated NLR has been associated with severe malaria and poor clinical outcomes, suggesting its potential as a prognostic indicator. Similarly, alterations in PLR can indicate platelet consumption and immune-mediated thrombocytopenia, which correlate with disease severity and complications. Given their potential to enhance malaria diagnostics, it is essential to thoroughly investigate the roles of NLR and PLR in the context of malaria. Understanding how these biomarkers correlate with disease severity and outcomes can improve risk stratification, guide therapeutic decisions, and ultimately enhance patient care. This review aims to consolidate current evidence on the diagnostic significance of NLR and PLR in malaria, identify gaps in research, and provide recommendations for their integration into clinical practice. By doing so, it seeks to contribute to the advancement of malaria diagnostics and support the global effort to combat this pervasive infectious disease.

## Methodology for review

This narrative review aimed to synthesize the existing literature on the diagnostic and prognostic significance of the NLR and PLR in malaria. The review process was structured to ensure comprehensive and rigorous selection of relevant studies while assessing the quality of the included articles. The methodology involved several key steps: literature search, inclusion/exclusion criteria, quality assessment, and data synthesis.

## Literature search strategy

A comprehensive literature search was conducted to identify relevant studies that investigated the diagnostic and prognostic role of NLR and PLR in malaria. The search was performed using a range of electronic databases, including:

- **PubMed**
- **Scopus**
- **Google Scholar**
- **Web of Science**
- **Cochrane Library**

The search strategy involved using keywords and medical subject headings (MeSH) terms such as: “Neutrophil-to-Lymphocyte Ratio,” “Platelet-to-Lymphocyte Ratio,” “Malaria,” “Diagnostic Biomarkers,” “Prognostic Markers,” and “Inflammatory Response in Malaria.” Boolean operators (AND, OR) were used to combine these terms to ensure comprehensive retrieval of articles related to the topic.

## Study selection process

The study selection followed a structured and rigorous process to ensure that only relevant studies were included in the review. The selection process was carried out in multiple stages:

**(a) Title and Abstract Screening**:

All retrieved articles were first screened based on their titles and abstracts to assess their relevance to the review topic. Studies that focused on the use of NLR and PLR in the context of malaria, including their role in diagnosis and prognosis, were retained for further examination.

**(b) Full-Text Screening**:

After the initial screening, the full texts of potentially eligible studies were reviewed. The studies that met the inclusion criteria were included in the final review. Any studies that were not directly related to malaria, did not assess NLR or PLR, or lacked data on diagnostic and prognostic significance were excluded.

**(c) Inclusion Criteria**:

The following inclusion criteria were applied to the studies:

- Published in peer-reviewed journals.
- Written in English.
- Investigated NLR and/or PLR as diagnostic or prognostic markers in malaria.
- Provided sufficient clinical or laboratory data to assess the relationship between NLR/PLR and malaria severity or prognosis.
- Included original research articles, clinical trials, cohort studies, or cross-sectional studies.

**(d) Exclusion Criteria**:

Studies were excluded based on the following criteria:

- Not directly related to malaria or its complications.
- Studies focusing on non-human subjects (e.g., animal models).
- Review articles, case reports, or editorials that did not provide original data.
- Studies that did not include NLR or PLR as primary or secondary outcome measures.
- Studies with insufficient or unclear methodology or data analysis.

## Quality assessment of included studies

To ensure the reliability and validity of the findings, a quality assessment was performed on all included studies. The quality assessment was conducted using standardized tools to evaluate the methodological rigor of the studies. The following approaches were employed:

**(a) Risk of Bias Assessment**:

The risk of bias was assessed using the **Cochrane Risk of Bias tool** for randomized controlled trials (RCTs) and the **Newcastle-Ottawa Scale (NOS)** for observational studies. Both tools assess potential biases related to the study design, participant selection, comparability of groups, and outcome reporting.

- **Cochrane Risk of Bias Tool**: This tool evaluates seven domains: random sequence generation, allocation concealment, blinding, incomplete outcome data, selective reporting, and other sources of bias.
- **Newcastle-Ottawa Scale (NOS)**: This scale assesses observational studies based on three broad categories: selection of study groups, comparability of groups, and the outcome evaluation. A higher NOS score indicates a lower risk of bias.

**(b) Study Design and Sample Size**:

Studies were evaluated for their design quality. RCTs and cohort studies were considered of higher quality due to their ability to establish cause-effect relationships. Cross-sectional studies and case-control studies were also included if they provided robust data on NLR and PLR in malaria patients. A minimum sample size of 50 participants was set as a benchmark for inclusion in the review to ensure statistical power.

**(c) Statistical Analysis and Data Reporting**:

The quality of statistical methods used in the studies was evaluated, including:

- Whether the sample size calculation was performed.
- Whether appropriate statistical tests were used to analyze the relationship between NLR/PLR and malaria outcomes.
- Whether the studies provided clear reporting of confidence intervals, p-values, and effect sizes to support their conclusions.

**(d) Consistency and Reproducibility of Findings**:

The consistency of findings across studies was assessed by evaluating whether similar trends in NLR and PLR values were reported across different malaria severity levels, geographic locations, and patient demographics. Studies that showed consistent and reproducible findings were given higher credibility.

## Malaria

Malaria is a life-threatening infectious disease that affects millions of people, particularly in tropical and subtropical regions^[10]^. It is caused by the Plasmodium parasite, which is transmitted to humans through the bites of infected female Anopheles mosquitoes. There are several species of Plasmodium that can cause malaria in humans, with *Plasmodium falciparum* and *Plasmodium vivax* being the most common and clinically significant. Malaria is primarily transmitted through the bite of infected mosquitoes. When an infected mosquito bites a person, it injects the malaria parasite into the person’s bloodstream. Malaria can cause a wide range of symptoms, which can vary in severity. Common symptoms include fever, chills, headache, and fatigue. Severe cases of malaria can lead to organ failure, anemia, and death. Malaria can present in different forms, including uncomplicated malaria with mild symptoms and severe malaria, which is a medical emergency. Severe malaria can cause complications such as cerebral malaria, severe anemia, ARDS, and multi-organ failure. Diagnosis of malaria typically involves blood tests, such as microscopy and RDTs, to detect the presence of the Plasmodium parasite in the patient’s blood^[11]^. Effective treatment for malaria usually involves antimalarial medications, which can vary based on the species of Plasmodium and the severity of the infection. Artemisinin-based combination therapies are commonly used for *P. falciparum* malaria. Malaria prevention strategies include the use of insecticide-treated bed nets, indoor residual spraying with insecticides, and chemoprophylaxis for travelers to endemic areas. In recent years, the development of a malaria vaccine called “RTS,S/AS01” has shown promise in reducing the risk of severe malaria in children in some regions. Malaria is a major global health problem, particularly in sub-Saharan Africa. It leads to significant morbidity and mortality, especially among children under the age of five and pregnant women^[12]^. Efforts to control and eliminate malaria include vector control programs, early diagnosis and treatment, and research into new drugs and vaccines. These efforts are part of a global initiative to reduce the burden of malaria and eventually eradicate the disease. Despite significant progress, malaria remains a major health challenge, and continued research and intervention efforts are needed to combat this disease effectively.

## Pathogenesis and clinical presentation of malaria

Malaria is a complex disease caused by Plasmodium parasites and transmitted through the bites of infected mosquitoes^[13]^. The pathogenesis and clinical presentation of malaria can vary depending on the species of Plasmodium involved, the immunity of the host, and other factors. Malaria begins when an Anopheles mosquito infected with Plasmodium bites a human^[14]^. The mosquito injects sporozoites into the person’s bloodstream during the bite. The sporozoites travel to the liver, where they invade hepatocytes. Inside hepatocytes, the sporozoites undergo a phase of asexual reproduction, leading to the formation of thousands of merozoites. Merozoites are released into the bloodstream and invade red blood cells (erythrocytes). Inside red blood cells, the merozoites undergo a cycle of asexual reproduction, causing the infected red blood cells to rupture. The ruptured red blood cells release more merozoites, which continue the cycle of invasion and replication. The clinical manifestations of malaria are primarily a result of the cyclical rupture of infected red blood cells and the release of merozoites into the bloodstream. These symptoms include: Recurrent, high-grade fever is a hallmark symptom of malaria. The timing of fever episodes varies depending on the Plasmodium species, with *P. vivax* and *P. ovale* causing a 48-hour cycle of fever (tertian malaria) and *P. falciparum* causing a 36- to 48-hour cycle (quotidian malaria). In severe cases, malaria can lead to complications such as cerebral malaria (infection of the brain), severe anemia, ARDS, multi-organ failure, and death. Immunity to malaria develops over time with repeated exposure, as individuals in endemic regions build up partial immunity to the disease^[15]^. This immunity can reduce the severity of symptoms and protect against severe forms of the disease. Pregnant women and children under five are particularly vulnerable to severe malaria. It’s important to note that the clinical presentation of malaria can vary, and severe cases can rapidly progress to life-threatening conditions. The specific manifestations and severity depend on factors such as the Plasmodium species involved, the individual’s immune status, access to healthcare, and the timeliness of diagnosis and treatment. Effective management of malaria includes early diagnosis and prompt treatment with appropriate antimalarial medications.

## Neutrophil-to-lymphocyte ratio (NLR)

The NLR is a simple and readily available marker used in medicine to assess the balance between two types of white blood cells in the bloodstream: neutrophils and lymphocytes^[16]^. It is a valuable indicator of the body’s inflammatory and immune response. NLR is calculated by dividing the absolute neutrophil count by the absolute lymphocyte count, which are both obtained from a standard CBC test. Neutrophils are a type of white blood cell (leukocyte) and are part of the innate immune system. They are the most abundant type of white blood cell and play a crucial role in the body’s defense against bacterial and fungal infections. Neutrophils are often the first responders to sites of infection and inflammation, where they engulf and destroy pathogens. Lymphocytes are another type of white blood cell and are part of the adaptive immune system. Lymphocytes include various cell types, such as T cells, B cells, and natural killer (NK) cells. Lymphocytes are primarily involved in immune responses against viral and parasitic infections, as well as in the regulation of immune reactions. NLR provides insights into the balance between the pro-inflammatory (neutrophils) and anti-inflammatory (lymphocytes) responses in the body^[17]^. An elevated NLR is often indicative of an increased inflammatory response. This can occur in various medical conditions, including infections, autoimmune diseases, and cancer. Changes in NLR can serve as a marker for the severity of an inflammatory condition and may suggest the presence of an underlying disease. NLR is widely used in clinical practice, particularly in oncology, where it is used to assess the prognosis and predict the outcomes of cancer patients. Elevated NLR is associated with poorer prognosis in various types of cancer. NLR is also used in assessing the severity of various inflammatory and infectious diseases, including sepsis, cardiovascular diseases, and autoimmune disorders. In the context of infectious diseases like malaria, NLR can be used to gauge the severity of the infection and monitor the body’s response to treatment.

## NLR in malaria

The NLR has been studied as a potential marker in the context of malaria to assess disease severity and the body’s immune response^[18]^. Elevated NLR is often observed in individuals with malaria, especially in cases of severe or complicated malaria. An elevated NLR indicates an increased inflammatory response in the body, which is a common feature of malaria infection. Neutrophils, as pro-inflammatory cells, tend to increase during infections. Elevated NLR values may be indicative of a more severe infection, as the body’s immune response is more pronounced in severe cases of malaria. NLR can serve as a marker for assessing the severity of malaria. Studies have shown that patients with higher NLR values tend to have more severe disease manifestations. It can help healthcare providers stratify patients based on disease severity and prioritize treatment for those with a higher likelihood of complications^[^19,20^]^. NLR values can be monitored over the course of malaria treatment. Changes in NLR may provide insights into the progression of the disease and the effectiveness of treatment. A decreasing NLR may indicate a positive response to treatment, whereas a persistently high or increasing NLR may suggest a lack of response or disease progression. Research studies have investigated the potential utility of NLR in differentiating between various Plasmodium species, such as *P. falciparum* and *P. vivax* malaria, and assessing the clinical course of the disease. NLR, along with other clinical and laboratory parameters, is used to guide treatment decisions and predict patient outcomes in cases of severe malaria.

## Platelet-to-lymphocyte ratio (PLR)

The PLR is another marker that, like the NLR, is used in medicine to assess the balance between different types of blood cells, specifically platelets and lymphocytes^[21]^. PLR provides insights into inflammation and immune response and can be relevant in various medical conditions, including infectious diseases like malaria. Platelets are small blood cells involved in the clotting process (hemostasis). They play a crucial role in stopping bleeding when blood vessels are injured. Lymphocytes are a type of white blood cell that plays a central role in the immune response. They include various cell types, such as T cells, B cells, and NK cells. PLR is calculated by dividing the absolute platelet count by the absolute lymphocyte count, both of which are obtained from a standard CBC test^[22]^. Elevated PLR values are often indicative of an increased inflammatory response, as platelets can become activated during inflammation. Changes in PLR may serve as a marker for the presence of an underlying disease or infection and its severity. In the context of malaria, PLR has been investigated as a potential marker to assess disease severity and the body’s immune response. Elevated PLR may occur in malaria, particularly in severe or complicated cases. This is often due to the inflammatory response associated with the infection. PLR can be used as a supplementary marker to assess the severity of malaria, monitor the progression of the disease, and guide treatment decisions. Like NLR, PLR is not used as a standalone diagnostic tool for malaria. Clinical evaluation, the presence of specific symptoms, and laboratory confirmation through tests like blood smears or RDTs are essential for diagnosis. PLR is typically used in conjunction with other clinical and laboratory parameters to provide a more comprehensive understanding of the patient’s condition and assist in making informed clinical decisions. The PLR is a marker of inflammation and immune response that can be relevant in assessing disease severity and progression, including in the context of malaria. While it has clinical utility, it is typically used in combination with other diagnostic and clinical information to aid in patient management^[^23,24^]^.

## Clinical implications of NLR and PLR in malaria management

The NLR and PLR are emerging as valuable biomarkers for diagnosing and predicting outcomes in malaria, particularly in resource-limited settings. These biomarkers are based on simple and widely available blood tests, such as the CBC, making them highly accessible in clinical practice. Below are some practical examples and case studies demonstrating how NLR and PLR could be used in clinical settings, especially where advanced diagnostic tools may not be readily available.

**(a) Early Detection of Severe Malaria in Resource-Limited Settings**

**Practical Example**: In a malaria-endemic region with limited access to sophisticated diagnostic tools, healthcare providers often rely on clinical symptoms (fever, chills, etc.) and basic blood tests to assess malaria severity. The use of NLR and PLR as supplementary markers can enhance early detection and prompt treatment, especially in settings where RDTs may be unavailable or unreliable.

**Case Study**: A 35-year-old male presents with fever, fatigue, and body aches in a rural healthcare facility in Sub-Saharan Africa. The local lab is equipped with a CBC machine but does not have access to RDTs or PCR testing for malaria. Upon reviewing his CBC results, the physician notices an elevated NLR of 6.2 (normal range: 1–3) and a reduced PLR of 105 (normal range: 150–250). Given these abnormal values, the healthcare provider suspects that the patient may be experiencing severe malaria and administers prompt antimalarial treatment. Later, microscopy confirms the diagnosis of *Plasmodium falciparum* malaria, and the patient recovers without complications.

**Clinical Implication**: In such settings, using NLR and PLR as adjunctive diagnostic tools can help identify severe malaria cases early, even in the absence of more expensive or complex diagnostic techniques^[19]^.

**(b) Monitoring Disease Progression and Prognosis**

**Practical Example**: Once a diagnosis of malaria is confirmed, NLR and PLR can be used to monitor disease progression. Elevated NLR levels can indicate ongoing inflammation and severe disease, while a decrease in PLR may reflect platelet consumption in severe malaria. These trends can be used to assess patient recovery or identify those at risk of complications, thus guiding treatment decisions.

**Case Study**: A 45-year-old woman with *Plasmodium falciparum* malaria is admitted to a local hospital in Southeast Asia. Initial tests show an NLR of 4.8 and a PLR of 120. After 48 hours of intravenous antimalarial treatment, a follow-up CBC reveals a drop in NLR to 3.1 and an increase in PLR to 145. Given these improvements in both biomarkers, the healthcare team is reassured that the patient is responding well to treatment and decides to transition her to oral antimalarial therapy.

However, for another patient in the same hospital, the NLR remains high at 7.5 and the PLR stays low at 90. This sustained elevation in NLR and low PLR prompt the physician to consider the possibility of complications like cerebral malaria or severe anemia, leading to a more aggressive management approach and closer monitoring in an intensive care unit (ICU).

**Clinical Implication**: NLR and PLR can help clinicians assess the effectiveness of treatment and detect complications early, which is particularly crucial in resource-poor settings where ICU access and intensive monitoring are limited^[20]^.

**(c) Identifying High-Risk Patients for Targeted Interventions**

**Practical Example**: In malaria-endemic areas with limited healthcare resources, prioritizing high-risk patients for intensive care and treatment can be challenging. NLR and PLR can assist healthcare workers in identifying individuals who are at greater risk for adverse outcomes, allowing for early intervention and more appropriate resource allocation.

**Case Study**: In a rural hospital in Sub-Saharan Africa, a 28-year-old pregnant woman presents with malaria symptoms. Her CBC reveals an NLR of 8.0 and PLR of 110. These values, coupled with her pregnancy, suggest that she may be at a higher risk for complications like anemia and preeclampsia. The healthcare team initiates early intervention, including intravenous antimalarials and close monitoring of both maternal and fetal health. The pregnancy is closely monitored with ultrasound, and the woman is discharged after recovery without any complications.

**Clinical Implication**: Pregnant women, children, and immunocompromised patients are particularly vulnerable to severe malaria. By using NLR and PLR to identify those at higher risk, healthcare providers can prioritize interventions and tailor treatment strategies, improving patient outcomes even in resource-constrained environments^[21]^.

**(d) Cost-Effectiveness in Resource-Limited Settings**

**Practical Example**: Malaria diagnostics can be expensive, especially in rural or under-resourced healthcare settings. NLR and PLR are simple, cost-effective markers that require only a CBC, a test commonly available even in low-resource environments. These biomarkers can reduce reliance on more expensive tests, making them a valuable addition to malaria management strategies in settings with limited access to advanced diagnostics.

**Case Study**: A small community clinic in rural India struggles with limited access to RDTs and microscopy due to cost constraints. The clinic implements the use of routine CBC testing, and abnormal NLR and PLR values are flagged as part of the diagnostic protocol. A patient presenting with fever and chills has an NLR of 5.6 and a PLR of 105. Based on these results, the clinic prioritizes the patient for malaria treatment and quickly confirms the diagnosis through microscopy. This approach reduces costs associated with expensive RDTs and ensures timely treatment.

**Clinical Implication**: The use of NLR and PLR can be a cost-effective way to enhance malaria diagnosis and treatment in settings with limited access to more advanced diagnostics, thus improving patient care and optimizing healthcare resources^[22]^.

**(e) Supporting Decision-Making in Malaria Elimination Programs**

**Practical Example**: Malaria elimination efforts require efficient and effective case management, particularly in low-transmission areas. NLR and PLR can be used to identify the severity of individual malaria cases, facilitating decision-making on whether to administer outpatient or inpatient care. These markers can help allocate resources more effectively, particularly in areas with limited healthcare infrastructure.

**Case Study**: In a malaria-elimination district in Latin America, a case of malaria is identified in a 60-year-old man who lives in a rural area with limited healthcare access. His CBC shows an NLR of 6.5 and PLR of 95, indicating the need for inpatient care and close monitoring. Given the location’s malaria elimination goals, the patient is transported to a larger facility for advanced treatment, while the healthcare team provides follow-up and community outreach to prevent further transmission.

**Clinical Implication**:For malaria elimination programs, NLR and PLR can assist in identifying patients who need more intensive care, ensuring that resources are allocated efficiently and that severe cases are not overlooked, even in low-transmission settings^[^23,24^]^.

**Limitations and Challenges Associated with NLR and PLR in Malaria**

**(a) Heterogeneity Across Studies:**
  - **Variation in Cut-off Values**: Different studies use varied cut-off values for NLR and PLR, which can lead to inconsistent conclusions regarding their diagnostic and prognostic significance.
  - **Differences in Malaria Species**: NLR and PLR may behave differently across different malaria species (e.g., *Plasmodium falciparum* vs. *Plasmodium vivax*), complicating their generalizability across populations infected with different species.
  - **Sample Size and Study Design Variability**: The small sample sizes and differences in study designs (cohort, case-control, cross-sectional) can lead to difficulties in extrapolating findings to larger or more diverse populations^[19]^.
**(b) Influence of Confounding Factors:**
  - **Co-morbidities and Infections**: Other concurrent infections or underlying conditions (e.g., bacterial infections, HIV, anemia) can influence NLR and PLR values, making it difficult to attribute changes solely to malaria.
  - **Age, Gender, and Nutritional Status**: Demographic factors such as age, gender, and nutritional status may impact inflammatory responses, leading to variability in NLR and PLR values.
  - **Medication Use**: The use of anti-malarial drugs or other medications (e.g., corticosteroids) can alter inflammatory markers like NLR and PLR, leading to misinterpretation of their true role in malaria severity^[20]^.
**(c) Limited Understanding of Mechanisms:**
  - **Pathophysiological Interpretation**: The exact mechanisms behind the alterations in NLR and PLR during malaria are not fully understood. While neutrophil and platelet responses are linked to inflammation, their specific role in malaria progression is not completely elucidated.
  - **Variability in Immune Responses**: The immune response to malaria can vary significantly among individuals based on genetic factors, immune history, and environmental influences, which complicates the interpretation of NLR and PLR as universal markers^[21]^.
**(d) Clinical Application Challenges:**
  - **Lack of Standardization**: There is no widely accepted protocol for the clinical use of NLR and PLR in malaria diagnosis and prognosis. This limits their integration into routine clinical practice, particularly in resource-limited settings.
  - **Need for Complementary Biomarkers**: NLR and PLR alone may not provide sufficient diagnostic accuracy or prognostic value. Their utility could be enhanced when used in combination with other biomarkers, which requires further research and validation.
  - **False Positive/Negative Results**: Changes in NLR and PLR are not specific to malaria and may be influenced by other conditions. As a result, relying solely on these biomarkers could lead to misclassification of patients, particularly in cases with non-malarial infections or other inflammatory conditions^[22]^.
**(e) Lack of Longitudinal Data:**
  - **Limited Follow-up Studies**: Most studies evaluating NLR and PLR in malaria are cross-sectional or retrospective in nature, providing only snapshot data on the relationship between these biomarkers and disease severity. Longitudinal studies are needed to better understand how NLR and PLR change over time and their ability to predict outcomes such as recovery, relapse, or mortality.
  - **Data on Dynamic Changes**: There is insufficient data on the dynamic changes in NLR and PLR during malaria treatment, which could provide more insights into their utility for monitoring treatment efficacy or predicting patient recovery^[23]^.
**(f) Resource and Technical Constraints:**
  - **Laboratory Access and Expertise**: While NLR and PLR are based on simple blood tests (e.g., CBC), access to these tests may be limited in rural or resource-poor settings, hindering their widespread use as diagnostic or prognostic markers for malaria.
  - **Technological Limitations**: The accuracy of automated hematology analyzers used for calculating NLR and PLR may vary depending on the technology and equipment used, which can affect the reliability of these biomarkers^[23]^.
**(g) Ethical and Cultural Considerations:**
  - **Patient Consent and Data Privacy**: In malaria-endemic areas, obtaining informed consent for studies may be challenging due to cultural and linguistic barriers. Ensuring ethical standards in the collection and use of biological samples is crucial for the validity and integrity of the research.

### Future directions

The future directions of NLR and PLR in the context of malaria research hold significant promise^[25]^. These ratios have shown potential as useful markers in understanding disease severity and the immune response in malaria. Future research should focus on validating the utility of NLR and PLR in different populations and regions where malaria is prevalent. Efforts should be made to establish standardized reference values for NLR and PLR, considering variations in baseline values among populations. Further research may lead to the development of simple and cost-effective diagnostic tools that incorporate NLR and PLR in malaria diagnosis. These tools could help healthcare workers in resource-limited settings to quickly assess disease severity and guide treatment decisions. Future studies could explore the potential of NLR and PLR in predicting specific complications of malaria, such as cerebral malaria or severe anemia^[^26,27^]^. Identifying patients at higher risk of complications could lead to more targeted and timely interventions. Research on NLR and PLR may contribute to the development of personalized treatment approaches for malaria. Identifying individuals with different immune responses could lead to tailored treatment strategies.

Combining NLR and PLR with other relevant biomarkers or clinical indicators can provide a more comprehensive understanding of the disease state^[28]^. Future research might focus on establishing multi-marker diagnostic or prognostic models for malaria. Researchers may explore the use of NLR and PLR to phenotype malaria infections, helping to distinguish between different Plasmodium species or strains. This could aid in more accurate diagnoses and targeted treatment approaches. Long-term, prospective studies are needed to track changes in NLR and PLR over time in malaria patients, from initial infection through recovery. This data can help identify patterns that provide insights into the dynamics of the immune response. The integration of NLR and PLR data into clinical decision support systems could assist healthcare providers in making informed treatment decisions. These systems could use NLR and PLR, along with other clinical information, to provide recommendations on the management of individual malaria cases. International collaboration and data sharing between researchers and institutions can accelerate progress in understanding the utility of NLR and PLR in malaria. Comparative studies across different endemic regions can provide valuable insights. The use of NLR and PLR in monitoring malaria trends and disease severity at the population level could inform public health interventions and policies. NLR and PLR have the potential to be valuable tools in the management and understanding of malaria^[27]^. As ongoing research and technological advancements continue to provide new insights, these ratios may become even more integral to malaria diagnosis, patient care, and public health efforts in the future.

## Recommendations for future studies on NLR and PLR in malaria

**(a) Standardization of Cut-off Values and Methodologies**:

Future studies should aim to establish standardized cut-off values for NLR and PLR across different malaria species, patient demographics, and disease stages. This will help ensure consistency and comparability across research findings and clinical applications. Standardization of laboratory techniques for measuring NLR and PLR, including the methodology for blood collection and processing, is necessary to minimize variability and improve the reproducibility of results.

**(b) Longitudinal and Multi-center Studies**:

Long-term, longitudinal studies are needed to assess how NLR and PLR evolve over the course of malaria infection and treatment. Such studies can provide insights into their predictive power for recovery, relapse, or mortality. Multi-center studies involving diverse populations from different geographical regions can help determine how environmental, genetic, and healthcare-related factors influence the utility of NLR and PLR in malaria diagnostics and prognosis.

**(c) Exploration of Mechanisms**:

More research is needed to explore the underlying biological mechanisms linking changes in NLR and PLR to malaria pathogenesis. Understanding how neutrophils, lymphocytes, and platelets interact during malaria infection will provide deeper insights into the role of these biomarkers in the immune response. Investigating the specific inflammatory pathways involved in malaria could clarify why NLR and PLR values fluctuate and how they relate to the severity of the disease.

**(d) Inclusion of Other Biomarkers**:

To enhance the diagnostic and prognostic accuracy of NLR and PLR, future research should investigate their utility when combined with other biomarkers, such as C-reactive protein, procalcitonin, or liver enzymes. This would help establish multi-biomarker panels for more accurate risk stratification and prognosis in malaria. Exploring the synergy between NLR/PLR and genetic markers or immune profiling could provide more personalized approaches to malaria management.

**(e) Evaluation in Different Malaria Species**:

Additional studies should compare the performance of NLR and PLR in *Plasmodium falciparum, Plasmodium vivax*, and mixed infections. Understanding whether these ratios perform similarly across different species will be critical for their widespread use in diagnosing malaria in various endemic regions. Further research should also explore how NLR and PLR respond to different malaria complications, such as cerebral malaria, severe anemia, or organ failure, and whether the ratios can predict these complications.

**(f) Investigation of the Impact of Co-Morbidities**:

Future studies should account for co-morbidities (such as HIV, tuberculosis, or bacterial infections) that could confound the interpretation of NLR and PLR in malaria patients. Research should explore whether NLR and PLR can be used to differentiate malaria from other febrile illnesses or inflammatory conditions. It would be important to assess how the presence of co-morbidities influences the diagnostic accuracy and prognostic value of NLR and PLR, potentially adjusting for these variables in future studies.

**(g) Improvement of Data Quality in Resource-Limited Settings**:

Given the importance of NLR and PLR as accessible, low-cost biomarkers, studies conducted in malaria-endemic, resource-limited settings should focus on evaluating the feasibility and practicality of using these biomarkers in routine clinical practice. Research should investigate the challenges of implementing NLR and PLR measurements in such settings, particularly in relation to laboratory infrastructure, access to trained personnel, and data quality.

**(h) Use of Advanced Statistical Models**:

Future studies could employ advanced statistical models (such as machine learning or artificial intelligence) to better understand complex relationships between NLR, PLR, and malaria severity. This approach could help identify novel predictive algorithms that incorporate these biomarkers along with clinical data. Exploring the potential of NLR and PLR as part of predictive modeling for early detection of malaria complications or mortality could be a focus of future research.

**(i) Ethical Considerations and Data Sharing**:

Researchers should ensure that studies involving NLR and PLR biomarkers adhere to ethical standards, particularly in areas with limited healthcare access. This includes obtaining informed consent and protecting patient privacy. Encouraging collaboration and data sharing among malaria researchers globally can facilitate meta-analyses and large-scale research efforts to validate the clinical utility of NLR and PLR.

Table 1 shows summary of the review on NLR and PLR in malaria (provided by the author).

**Table 1:** Showing Summary of the Review on Neutrophil-to-Lymphocyte Ratio (NLR) and Platelet-to-Lymphocyte Ratio (PLR) in Malaria.

Aspect	Summary
**Objective of the Review**	To evaluate the diagnostic and prognostic significance of NLR and PLR in malaria
Key Findings on NLR	Higher NLR values (typically >4.0) associated with severe malaria and poor outcomes.
	NLR correlates with parasitemia levels and complications like organ failure and anemia.
	Elevated NLR serves as an early indicator of disease progression and severity
Key Findings on PLR	Lower PLR values (typically <130) associated with severe malaria.
	PLR inversely correlates with platelet count, reflecting the inflammatory response in malaria.
	PLR is a prognostic marker, with lower values linked to higher mortality and prolonged recovery
Diagnostic Value	NLR and PLR serve as cost-effective and easily accessible biomarkers for diagnosing malaria severity.
	Both ratios may assist clinicians in distinguishing between mild and severe malaria cases
**Prognostic Value**	Elevated NLR and reduced PLR can predict poor outcomes, including higher mortality and longer hospital stays
**Clinical Implications**	Integration of NLR and PLR into routine clinical practice could enhance early detection and risk stratification in malaria management
Recommendations for Future Research	Standardization of NLR and PLR cut-off values across different malaria species and populations is needed
	Further studies should explore the combined use of NLR and PLR with other biomarkers for improved diagnostic accuracy

## Conclusion

The NLR and PLR have emerged as promising hematological biomarkers in the diagnosis and management of malaria. Their ability to reflect systemic inflammation and immune response dynamics provides valuable insights that extend beyond traditional diagnostic methods. NLR and PLR offer cost-effective, easily accessible metrics that can be derived from routine CBC results. Elevated NLR levels are associated with severe malaria and poor clinical outcomes, indicating a dysregulated immune response characterized by heightened neutrophil activity and lymphocyte depletion. Alterations in PLR suggest platelet consumption and immune-mediated thrombocytopenia, correlating with disease severity and complications. Integrating these ratios into clinical practice could provide complementary information to existing diagnostic methods like RDTs and microscopy, facilitating timely and accurate risk assessment and treatment decision-making.

## Data Availability

Not applicable as this a review.

## Research registration unique identifying number (UIN)

Not applicable as this a review.
